# The Effect of Bonding Surface Design on Shear Bond Strength of 3D-Printed Orthodontic Attachments

**DOI:** 10.1155/2023/6697178

**Published:** 2023-08-07

**Authors:** Saeed Noorollahian, Zahra Zarei, Leila Sadeghalbanaei, Khashayar Pakzamir

**Affiliations:** ^1^Department of Orthodontics, Dental Implants Research Center, Dental Research Institute, School of Dentistry, Isfahan University of Medical Sciences, Isfahan, Iran; ^2^Department of Orthodontics, Dental Research Center, Dental Research Institute, School of Dentistry, Isfahan University of Medical Sciences, Isfahan, Iran; ^3^Department of Orthodontics, Dental Materials Research Center, Dental Research Institute, School of Dentistry, Isfahan University of Medical Sciences, Isfahan, Iran; ^4^Private Practice, Isfahan, Iran

## Abstract

**Introduction:**

This study compared the shear bond strength (SBS) of four innovative designs of the bonding surface of 3D-printed orthodontic attachments with conventional mesh design.

**Methods:**

In this in vitro study, the bonding surface design in different groups was as follows: Group 1, flat surface without any feature as a negative control; Group 2, concentric circles with no cuts; Group 3, concentric circles with 16 radial cuts; Group 4, concentric circles with 32 radial cuts; Group 5, small cones with a flat end and rounded edges; Group 6, mesh-based commercially available metal brackets of the maxillary central incisor (standard edgewise, Dentaurum®) as a positive control (*n* = 20). In Groups 1–5, attachments were designed with SolidWorks® Software and printed with a 2K DLP-LCD printer with hard tough resin (eSun®). All the samples were bonded to the restorative composite resin (Solafil®) surfaces with orthodontic composite resin (CuRAY-ECLIPSE®). The samples were examined for SBS with a universal testing machine after thermocycling (1,000 cycles of 5‒55°C). Data were analyzed with Shapiro–Wilk, one-way ANOVA, and Bonferroni tests. The statistical significance level was set at 0.05.

**Results:**

The mean SBS was significantly different between all the groups (*P* < 0.001) except for Groups 2 and 5 (*P* = 1.00) and Groups 2 and 6 (*P* = 1.00). Group 4 had the highest mean of SBS.

**Conclusion:**

The bonding surface design significantly influenced the SBS of orthodontic attachments. The concentric circles with 32 cuts had higher bond strength than other designs and can be suggested as a new bonding surface design for orthodontic attachments.

## 1. Introduction

The bond strength of attachments is a critical factor in the clinical success of orthodontic treatments. It depends on various factors, such as the tooth surface preparation, the adhesive, the bonding process, and the characteristics of the attachment [1–4].

The attachment bonding surface (ABS) design plays a critical role. Most attachments do not have a chemical bond with the adhesives, so many efforts have been made to improve mechanical retention [5–7].

The actual bonding surface area has been proposed as an important variable in mechanical retention and bond strength. The macroscopic and microscopic increase of this surface can improve the bond strength. The macroscopic area of the bonding surface has esthetic and hygienic limitations [8]. Reducing the macroscopic surface makes ABS design more crucial to maintaining and increasing the microscopic surface [9]. Therefore, various ABS designs have been introduced to improve the bond strength, including retentive undercuts or grooves, brazed or welded mesh wires, laser structured undercuts, and metal or ceramic particles added to the bonding surface [10–12]. Many studies have investigated the effect of ABS design on the shear bond strength (SBS) of attachments. The variables assessed in these studies include different bonding surface designs and shapes, mesh density, mesh gauge, and the width and depth of the mesh spaces [12–19].

Three-dimensional (3D) printing is a new technology that makes it possible to create structures with complex shapes. The increased accuracy, ease of design and manufacturing, high-production speed, the possibility of using various materials, and reduced costs are some of its advantages [20]. This study evaluated the SBS of 3D-printed resin attachments with different ABS designs and compared them to the conventional mesh base designs.

## 2. Materials and Methods

### 2.1. 3D Designing and Printing

**Figure 1:**
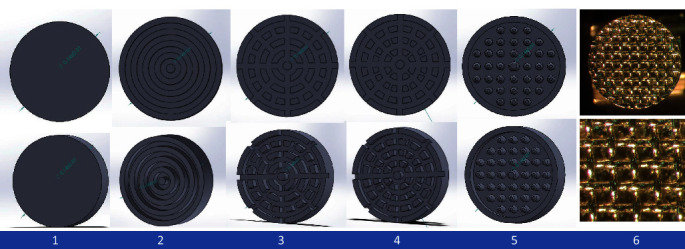
Bonding surface designs of attachments. (1) Flat surface without any features, (2) concentric circles with no cuts, (3) concentric circles with 16 cuts, (4) concentric circles with 32 cuts, (5) anchor pylon, and (6) mesh-based metal attachment.

In this in vitro study, 120 samples were tested in six groups (*n* = 20). The bonding surface designs in different groups were as follows: Group 1, flat surface without any feature as a negative control; Group 2, concentric circles with no cuts; Group 3, concentric circles with 16 radial cuts; Group 4, concentric circles with 32 radial cuts; Group 5, small cones with a flat end and rounded edges (0.05 mm fillet, 5° draft); and Group 6, mesh-based metal brackets of the maxillary central incisor (standard edgewise, Dentaurum®, Ispringen, Germany) as a positive control. The shape of the bonding surface in all the groups was a 3.5 mm diameter flat circle.  Figure 1 shows the bonding surface designs of different groups.

**Figure 2:**
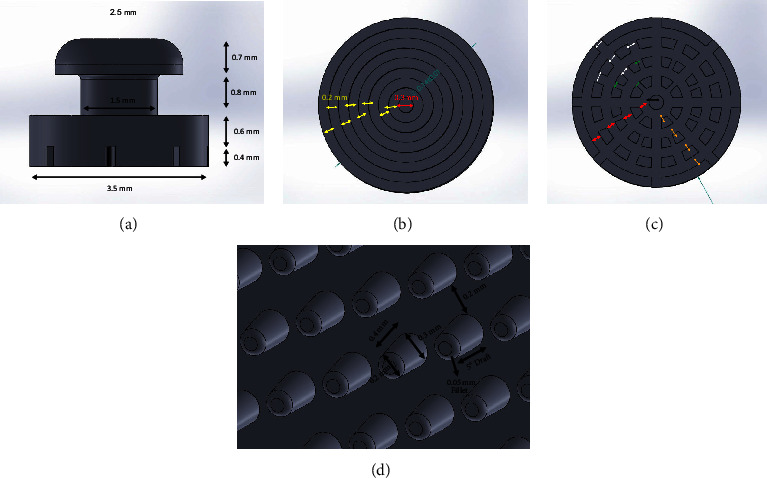
Dimensions of 3D-printed attachments: (a) outer dimensions. (b), (c), and (d) Dimensions on the bonding surface in Group 2 (b), Group 4 (c), and Group 5 (d). Red, white, and yellow arrows = 0.2 mm, black arrow = 0.3 mm, and green arrows = 0.1 mm.

**Figure 3:**
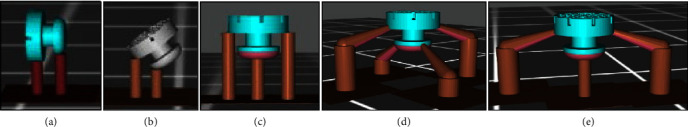
Different kinds of support mountings (a–e).

In Groups 1–5, attachments were designed similar to lingual buttons with Solidworks® software (Dassault Systèmes SolidWorks Corporation, Waltham, Boston, USA). The dimensions are shown in Figure 2(a)–2(d). In pilot printing, different attachment orientations and different kinds of support mountings were tested, and finally, linear 3-support mounting was selected (Figure 3(a)– 3(e)). The samples were printed with a 3D resin printer (2K DLP-LCD, Tivan Ganjineh Fanavaran Co, Tehran, Iran) with hard tough resin (eSUN, Shenzhen, China). The thickness of each print layer was 20 *µ*m. Then they were washed with 70% isopropyl alcohol to remove the uncured resin. To complete curing and obtain maximum hardness, the samples were irradiated by 20 UV lamps (1,000 mA intensity and 3.7 V voltage) for 12 min from four directions simultaneously and at the closest possible distance.

### 2.2. Bonding Process

Cubic molds with 5 × 5 × 4 mm dimensions were milled in transparent polycarbonate ingots at a 5 mm distance from each other. The restorative composite resin (Solafil, Trent Dent Co, London, UK) was packed into the cavities in 2 mm layers. Each layer was light cured for 20 s from the closest possible distance with a light-curing device (OrthoLux, 3 M Unitek, St. Paul, Minnesota, USA) with a power of 450 mW/cm^2^. To simulate clinical conditions and achieve similar smoothness on all surfaces, the outer surface of light-cured composite resin bulks was trimmed with the fine plate of a model trimmer (Dual wheel Model Trimmer V230, Dentaurum, Ispringen, Germany) with the water flow as coolant. The surface of the composite resins was etched with 37% phosphoric acid gel (CuRAY-ECLIPSE, Sci-PHARM, Pomona, California 91768, USA) (30 s), washed with water (15 s), and dried with oil-free airflow (10 s). The surface was coated with unfilled resin (CuRAY-ECLIPSE, Sci-PHARM, Pomona, California 91768, USA) and thinned with gentle airflow. Orthodontic composite resin adhesive (CuRAY-ECLIPSE, Sci-PHARM, Pomona, California 91768, USA) was used to bond attachments on the above-mentioned composite resin surface. Curing was performed for 20 s from two opposite sides (40 s in total) [21] at the closest distance with a light-curing device (OrthoLux, 3M Unitek, St. Paul, Minnesota, USA) with a power of 450 mW/cm^2^.

All the samples were kept in distilled water at 37°C for 24 hr and then thermocycled 1,000 times at 5‒55°C (Delta Tpo2, Nemo co, Mashhad, Iran) with a 20 s immersion time.

### 2.3. Shear Bond Strength (SBS) Assessment

The SBS of the samples was measured by a universal testing machine (K-21046, Walter + bia, Iohningen, Switzerland). The force was applied by a beveled flat-end metal rod at the attachment‒composite resin interface with 1 mm/min crosshead speed, parallel to the bonding surface [21].

The data were analyzed by SPSS (Version 22, SPSS Inc, Chicago, USA). The Shapiro–Wilk test was used to assess the normality of data distributions. One-way ANOVA was run to compare the groups, and Bonferroni tests were applied for pairwise comparisons of the groups. The significance level was set at 0.05.

## 3. Results

**Figure 4:**
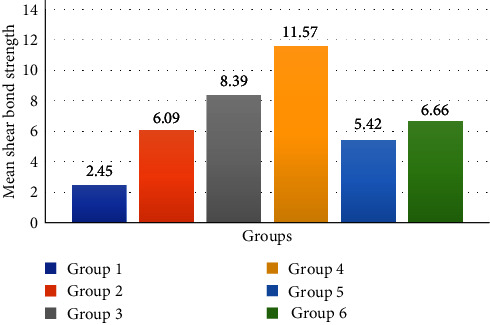
Mean shear bond strength of Groups 1–6.

The Shapiro–Wilk test showed that the data had a normal distribution (*P* < 0.001). The one-way ANOVA test showed statistically significant differences between the groups (*P* < 0.001). Two-by-two comparisons of the groups using the Bonferroni test showed a significant difference between the groups (*P* < 0.001) except for Groups 2 and 5 (*P* = 1.000) and 2 and 6 (*P* = 1.000). The data are shown in Table 1. The comparison of mean SBS of all six groups is demonstrated in Figure 4.

## 4. Discussion

Sufficient bond strength of orthodontic bonded attachments is necessary for clinical success, and their accidental debonding leads to unplanned appointments, prolonged treatment duration, and increased costs [22]. In many studies, changes in parameters, such as tooth surface preparation techniques, type of adhesive, bonding techniques, and the ABS design, have been modified to improve bond strength [1].

Increasing the acid concentration and etching time leads to irreversible enamel structure damage and greater susceptibility to decalcification and caries [23].

The main mechanism of attachment bonding is the mechanical retention of adhesive tags in the ABS undercuts. Any attempt to increase effective ABS undercuts increases the attachment bond strength [5].

Many studies have evaluated the bond strength of orthodontic attachments with different ABS designs [13–17], demonstrating that ABS design significantly affects the SBS, consistent with the present study.

In Group 1 (negative control), the attachments with a completely flat bonding surface without any undercuts showed 2.45 ± 0.79 MPa bond strength, possibly due to a chemical bond between the hard tough resin used in 3D-printed attachments and the adhesive resin. Therefore, this material can be a good choice for fabricating orthodontic bonding attachments because this chemical bond will improve the bond strength potential. The bond strength of this group was significantly lower than mesh-based metal attachments (6.66 ± 1.33), which merely have mechanical retention. This means it is also necessary to have effective undercuts for mechanical retention in designing the bonded orthodontic attachments with this material.

The bond strength of attachments with concentric circles design (Group 2) was not significantly different from attachments with anchor pylon design (Group 5) and mesh-based metal brackets (Group 6). In Groups 2 and 5, the hard tough resin material had a chemical bond to the adhesive, but in Group 6, the metal mesh had no chemical bond. Therefore, we can conclude if these three ABS designs are fabricated from the same material, the mesh design will have a higher bond strength.

In concentric circles without cuts, the lack of sufficient escape way for the adhesive, more air trapped air, less resin penetration into the undercuts, and less effective contact area (ECA) between adhesive and ABS might be the reason.

The concentric circles' design (Groups 2, 3, and 4) may increase the possibility of uniform stress distribution between the ABS and adhesive [24]. Providing a passage for more air escape causes better adhesive penetration into the undercuts and a more real contact area between the adhesive and attachment [9, 15]. For this purpose, radial cuts were added to the concentric circles in Group 3 (16 cuts) and Group 4 (32 cuts), which showed significant improvements in bond strength. Group 4 (concentric circles with 32 cuts) had the highest SBS due to more cuts.

In Group 5, the bonding surface of the attachments consisted of small cones with rounded edges (anchor pylon design). In this group, the bond strength was lower than in Group 6 (metal mesh-based). Atashi et al. [16] and Gibas et al. [17] reported that anchor pylon had greater SBS than the mesh design. This controversy is probably related to the different dimensions of the cones, the type of adhesive, and the attachment material. The SBS of this group was significantly lower than Groups 3 and 4, probably due to the more ECA between the adhesive and attachment in Groups 3 and 4.

The minimum accepted SBS of orthodontic bonded attachments for clinical success has been suggested in the range of 5.9‒7.9 MPa [25]. In the present study, all the 3D-printed groups fulfilled this requirement except the negative control group, whose bonding surface was completely flat without undercuts.

The smaller the dimension of spaces as undercuts, the higher the potential of ECA between the adhesive and ABS; in addition, the deeper the adhesive penetration into undercuts, the higher the ECA and SBS. Adhesives with higher filler content have better mechanical properties and can provide more SBS. However, they have more viscosity and less flow into fine spaces, which can reduce ECA and SBS. Therefore, it is important to consider adhesive resin viscosity, dimension, and pattern of undercuts [14, 19].

The width of grooves in Groups 2, 3, and 4 was 200 *µ*m. This dimension was considered, according to the previous studies, as a suitable space for a better inflow of orthodontic adhesives. Merone et al. [24] reported that 150 *µ*m mesh spacing showed greater SBS than 100 *µ*m ones. Liu et al. [26] also showed that 200 *µ*m spaces had higher SBS than 100- and 150 *µ*m ones.

Restorations and adhesives undergo thermal changes in the oral environment, affecting the bond strength [27]. Intraoral temperature changes have been reported in the range of 0‒65°C [28]. The protocol that the International Organization for Standardization (ISO) proposed in 2015 for thermocycling is 500 cycles at 5‒55°C, with 20 s of dwell time and 5‒10 s for transfer at room temperature. The 500 cycles simulate a period of <2 months which is too short compared to clinical conditions. For this reason, in this study, all the samples were thermocycled 1,000 times at 5‒55°C to better simulate oral conditions.

Since shear forces in the oral cavity are the common cause of debonding of attachments, evaluation of SBS is more common in studies [29]. Therefore, this study used this method to investigate bond strength values.

In many studies, extracted teeth are used to evaluate the bond strength of attachments; however, other variables have also been incorporated into the studies due to variations in the surface enamel quality and curvature. For this reason, the same composite blocks were used in the present study for the homogeneity of the bonding substrate. Since the bond failure usually occurs at the adhesive‒ABS interface, it is possible to generalize the results of the present study to the enamel surface bonding. The same method has been used in previous studies [7, 30, 31].

Samples in Group 6, as a positive control, had a flat circle shape ABS with a similar diameter to samples in Groups 1–5. These eliminated extra variation due to ABS shape differences and facilitated result analyses.

3D printing technology allows producing complex shapes with different materials. Using this method to manufacture samples reduced the confounding factors and increased the study's accuracy. The relatively low-standard deviation in each group in the present study confirms this.

Polyurethane polymer resins are biocompatible and used in medical applications such as dental aligners and artificial hearts. Aliphatic urethane acrylate is a light-sensitive resin with low viscosity and favorable mechanical properties [32, 33] The hard tough resin used in this study contained 40%‒50% urethane aliphatic acrylate compounds, 20%‒40% monomer, 3%‒5% light activator, and 2%‒5% pigment with favorable mechanical properties. The lack of failure in the body of the attachment base of tested samples during the SBS assessment indicates that this resin has sufficient mechanical properties and can be suggested to make orthodontic attachments.

Despite attempting to simulate the conditions of the present study to the circumstances of the oral environment, many variables could still affect the results. The various factors that have a significant influence on bond strengths should be pointed out more intensely. In fact, saliva [34], blood [35], bleaching agents [36], or other contaminants have been demonstrated to have a significant influence on bond strength. Therefore, these variables should also be considered carefully in combination with laser pretreatment in future clinical and laboratory tests.

## 5. Conclusion

Within the limitations of this study, it can be concluded that:

1. The design of the bonding surface of orthodontic attachments significantly affects their bond strength.
2. Due to the chemical bond with adhesive resin and sufficient strength, the hard tough resin can be used as a suitable material for 3D-printed orthodontic bonding attachments.
3. 3D-printed attachments with hard tough resin and concentric circles with 200 *µ*m width spaces on bonding surface design, even in simpler designs, provide bond strength equivalent to mesh-based conventional metal attachments.
4. The concentric circles with 32 radial cuts showed more bond strength than other designs. Therefore, they can be suggested as a new bonding surface design to provide optimum bond strength in orthodontic attachments.

## Figures and Tables

**Table 1:** Shear bond strengths in different groups (MPa) (*n* = 20).

Groups	Min	Max	Mean (SD)	Confidence interval (95%)	
				Lower bond	Upper bond
1	1.28	3.75	2.45 (0.79)	2.08	2.82
2	4.12	7.62	6.09 (1.02)^ab^	5.61	6.57
3	6.40	10.49	8.39 (1.37)	7.74	9.03
4	9.39	13.25	11.57 (1.18)	11.01	12.12
5	3.83	7.32	5.42 (1.2)^a^	4.86	5.98
6	4.25	8.93	6.66 (1.3)^b^	6.03	7.29

1: Flat surface without any feature (negative control group). 2: Concentric circles with no cuts. 3: Concentric circles with 16 cuts. 4: Concentric circles with 32 cuts. 5: Anchor pylon. 6: Mesh-based metal attachment (positive control group). Letters a and b show insignificant differences.

## Data Availability

The strength test reports data and SPSS output data used to support the findings of this study are available from the corresponding author upon request.
